# Ultrasound Study to Validate the Anterior Cervical Approach to the Longus Colli Muscle Using Electromyography Control Alone

**DOI:** 10.5334/tohm.545

**Published:** 2020-09-29

**Authors:** Lucy A. Hicklin, Serdar Kocer, Natalie A. Watson, Marie-Helene Marion

**Affiliations:** 1 Department of Otolaryngology, St George’s University Hospital NHS Foundation Trust, GB; 2 FMH Physical Medicine and Rehabilitation, Hôpital du Jura, Porrentruy, CH

**Keywords:** longus colli injection, anterocollis, anterior median approach, EMG guided injection, Ultrasound cervical muscles

## Abstract

**Background:**

One of the main difficulties in the treatment of dystonic anterocollis is the injection of the deep flexor muscles of the neck such as Longus Colli (LCo). The injection of the LCo has been regarded as difficult and potentially dangerous; since we published our anterior median approach, a number of questions about the precision and the safety of our technique have been raised by colleagues.

**Methods:**

7 patients with anterocollis were injected, using our injection technique and when the needle was deemed to be in place, we used the ultrasound probe to determine what the needle had passed through, the depth of the tip of the needle and if the identified muscle was indeed LCo.

**Results:**

On the ultrasound section the LCo muscle is between 24 and 28 mm deep in the patients examined in this study. The location of the needle was confirmed by ultrasound and in most cases the needle was placed in the right axis but sometimes not deep enough. The EMG control made it possible to correct the depth in all cases. In most of the injections the needle traversed the thyroid. No acute incident occurred by this route of injection. Injections were performed between 22 and 28 mm deep.

**Discussion:**

From this study and based on a review of complications over 9 years experience with injecting LCo under EMG control using an anterior approach, we conclude that this technique is precise, safe and well tolerated.

**Summary (Highlights):**

The injection of the Longus Coli muscle for anterocollis has been regarded as difficult and potentially dangerous. This study showed, using ultrasound to determine the needle trajectory, that the anterior approach using EMG control is a precise, safe and well tolerated technique.

## Introduction

Anterocollis is one of the most challenging dystonic postures to correct and is a common reason for treatment failure [1,2,3]. One of the main difficulties in the treatment is the injection of the deep flexor muscles of the neck such as longus colli (LCo) and longus capiti (LCa) [1,2,4,5]. The injection of the LCo has been regarded as difficult and potentially dangerous [6] and so it is not routinely offered in the clinical setting. There are sporadic case reports [8,9,10] and a short series in the literature describing injection of the longus colli under fluoroscopy [7], CT [8], or ultrasound [9,10] guidance, sometimes under sedation [7]. The majority of descriptions [7,8,9] describe a lateral approach taking the injector close to the carotid sheath. Injection with imaging and/or anaesthetic is both more time consuming and expensive than the pure electromyography (EMG) technique.

**Figure 1:**
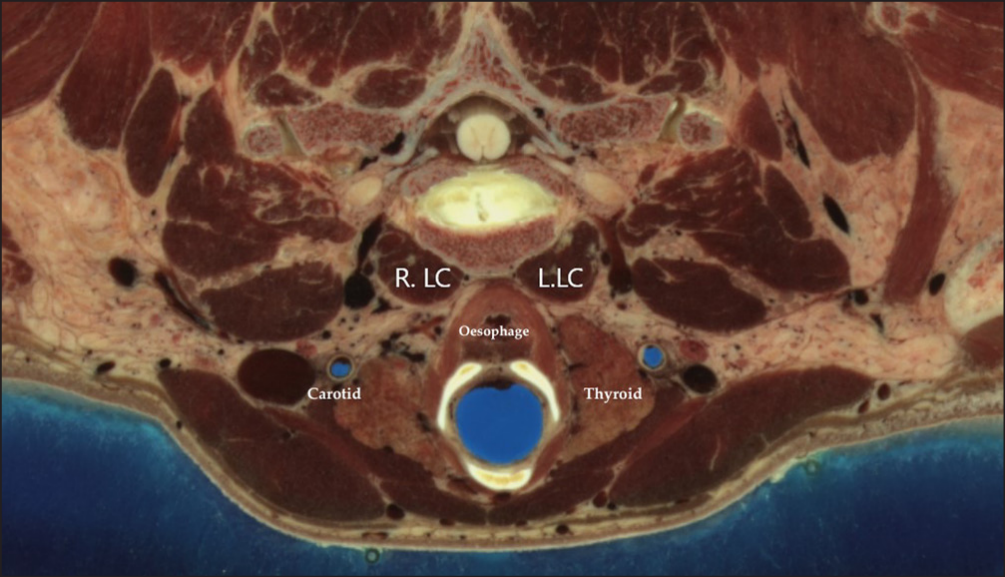
Anatomical structure at the level of C5–C6. R. LC: right Longus Collii, L.LC Left Longus Collii. Oesephagus. Thyroid gland. Carotid artery. From “The visible human project” of the National Library of Medicine, Bethesda, USA https://www.nlm.nih.gov/research/visible/visible_human.html.

In our joint Neurology-ENT clinic we have been successfully injecting patients with anterocollis since May 2010. We published in an early report in 2011 on the technique of injection of the LCo [4], our approach uses EMG to identify the dystonic muscle and by approaching the LCo via the avascular corridor [11,12] bordered by the anterior border of the sternocleido-mastoid (SCM) and lateral border of the trachea (Figure 1).

We have performed a total of 131 injections in 28 patients over a 9 year period. After publishing our approach in 2011 [4] we have had discussions with other injectors in the field, which have produced a number of questions about the precision and the safety of our technique.

1. Are we actually targeting the LCo muscles?
2. Are we at risk of damaging vascular structures?
3. Are we causing dysphagia and is this technique safe?
4. Do we traverse the thyroid gland during the injection?

To address these questions we studied a number of our patients performing our normal EMG protocol with the addition of ultrasound (US) confirmation of the position of the needle once in place. We have also reviewed the notes of our patient cohort to look for complications.

## Method

7 patients were selected from the treatment cohort on the basis that they were having LCo injections for cervical dystonia (anterocollis) and were due for re-injection. We were not able to study the whole cohort due to constraints of availability of the study team and ultrasound equipment which we do not routinely use in our clinic.

The case notes of all our patients undergoing LCo injections were reviewed; the total number of injections and any complications were noted.

The LCo is a long slender muscle consisting of three interconnecting parts. Firstly, the lower oblique runs upwards from T2 to T3 vertebral bodies inserting on the C5–C6 anterior tubercles. Secondly, the upper oblique arises from the C3 to C5 anterior tubercles, inserting on the atlas. Lastly the vertical part arises from the C5 to C7 and T1 to T3 vertebral bodies, inserting on the C2–C4 vertebral bodies. It is deep to the prevertebral fascia, the trachea and oesophagus are medial, and the carotid sheath is lateral.

### Injections

(Video) Patients are directed to lie supine on the couch, when lying down the neck muscles are at rest which means that spontaneous EMG activity will be from the target muscles. The EMG leads are attached, 120 units Abobotulinumtoxin A is drawn into a 1 ml syringe and attached to the EMG needle (myoject 37 mm/27 g). The neck is prepared with chlorhexidine wipes. No local anaesthetic is necessary. We inject LCo in 2 places on each side with 30 units per site, so 60 units Abobotulinumtoxin into the right and into the left LCo; the higher level is around C6 ensuring that it is below the cricopharyngeus to avoid dysphagia, and another point approximately 2 cm below that level. The distance between the injections is dictated by the length of the neck and the severity of the anterocollis posture. The left sided injection is approached standing on the right side of the patient and directing the needle from below upwards at an angle of about 30 degrees. Placement of the needle in LCo is confirmed by asking the patient to raise the head off the bed and bend forwards to put the chin on the sternum. The patient is also asked to swallow to ensure little or no activation of the cricopharyngeus. The cricopharyngeus has a constant tone and there is a brief hiatus representing relaxation when swallow is triggered so by asking the patient to swallow we can listen for this pattern; if there is significant activation the needle is withdrawn and re-inserted lower down. The right-sided injections are approached from the left side of the patient inserting the needle from above downwards, again with a 30 degree trajectory.

**Video:** **Left Longus Colli muscle injection** Injection of a patient with anterocollis of the left Longus colli muscle under EMG guidance.

With the retraction of the trachea one is very close to the longus colli and directly above it with only the cervical spine behind as long as the needle is kept parallel to the trachea, other structures are not at risk.

### Ultrasound study

**Figure 2:**
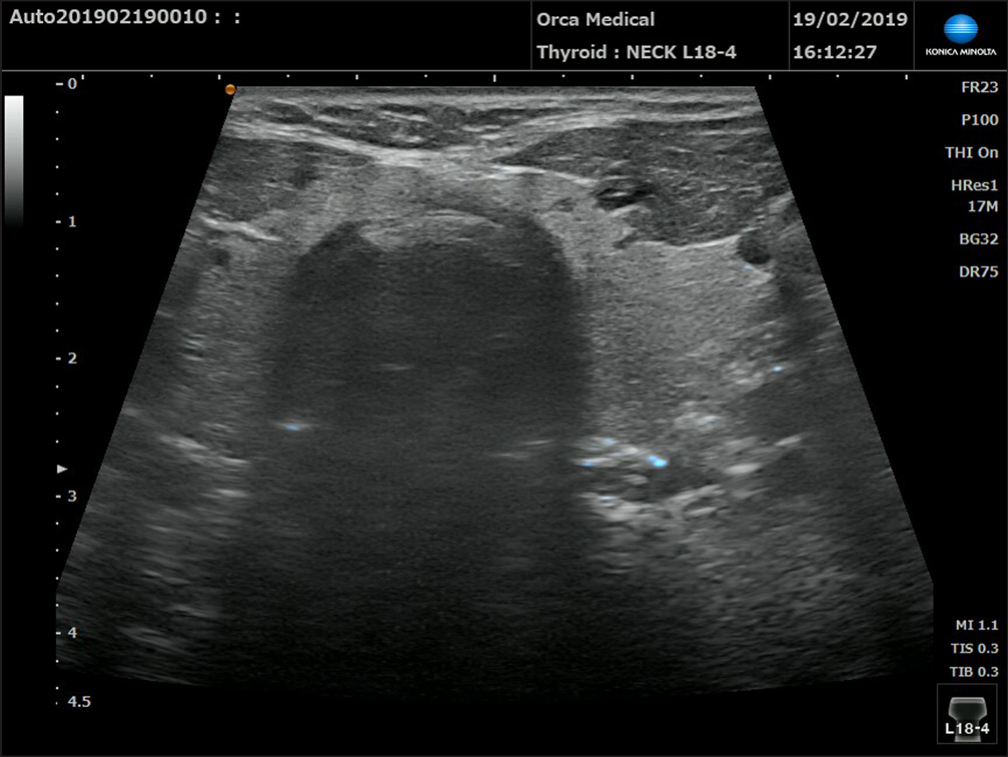
Position of the needle on ultrasound view. Blue dot on the left side representing the tip of the EMG needle, going in the Longus Colli muscle through the thyroid gland (SNV mode). Small cysts, benign in appearance, can be seen in the thyroid.

**Figure 3:**
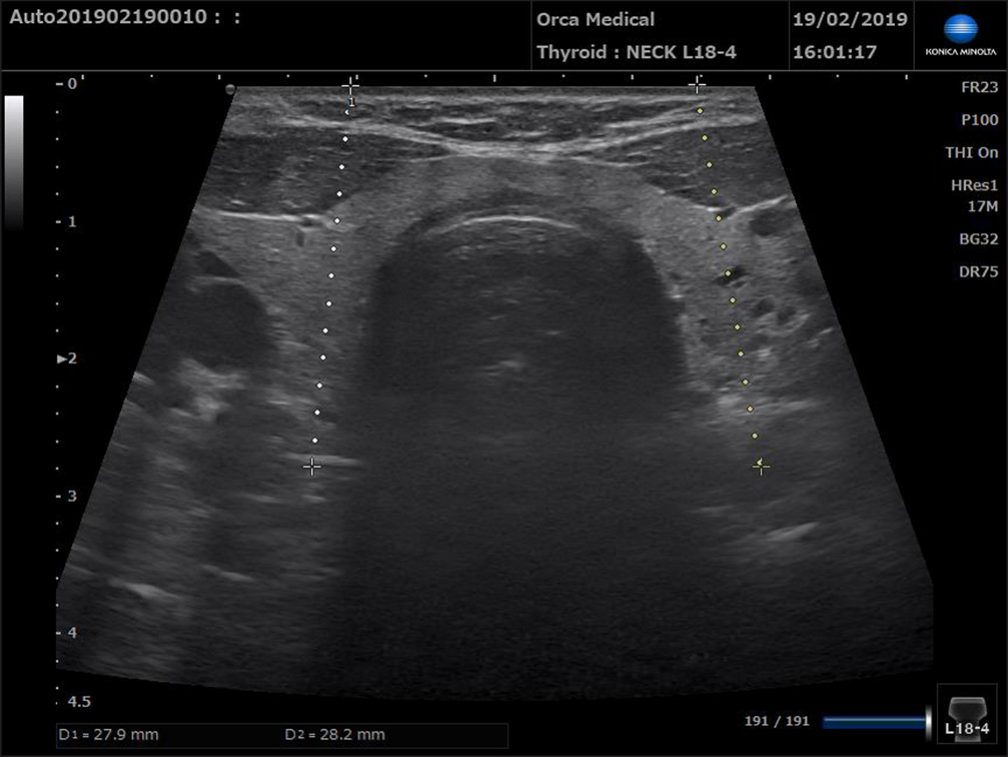
The distance between the skin and the muscle belly of the Longus Colli at the level of C5–C6. 27.9 mm on the right side and 28.2 mm on the left side Small cysts, benign in appearance, can be seen in the thyroid.

Once, using the above protocol, the needle was deemed to be in place we used the ultrasound probe to determine what the needle had passed through, the depth of the tip of the needle and if the identified muscle was indeed LCo. Still pictures were saved for further analysis (Figures 2–3). The study was performed with Konica Minolta sonimage sonography HS1 equipped with a linear high frequency probe 4–18 megahertz. SNV mode was used to facilitate identification of the needle. The blue dot visible on some images corresponds to the tip of the needle (with the help of SNV mode) (Figure 2). On the supine patient, the 4 cm long ultrasound transducer was placed in the medial and transverse position at the C5 C6 vertebral body on the anterior aspect of the neck between the thyroid cartilage and the sternum [13]. Using this position [14,15], we were able to see the larynx with the carotid arteries, jugular veins on either side, oesophagus on the left side and the thyroid and deep to the thyroid the LCo muscles. (Figures 2–3).

## Results

On the section used which goes through the middle of the neck the LCo muscle is between 24 and 28 mm deep in the patients examined in this study. When the LCo muscle image was not good enough to identify the needle inside, the probe was slightly displaced laterally. With the probe in this position, the muscle is closer to the probe and in this case the depth of the LCo muscle was on average 22 mm in our patients. The upper and lower injection sites are separated by a mean of 10–20 mm.

The location of the needle was confirmed by ultrasound and in most cases the needle was placed in the right axis but sometimes not deep enough. The EMG control made it possible to correct the depth in all cases. In most of the injections the needle traversed through the thyroid. No acute incident occurred by this route of injection. Injections were performed between 22 and 28 mm deep. In one patient with a long neck the lower point did not cross the thyroid. Traction of the larynx probably helps the operator to find landmarks but generally does not allow widening of the pathway of the needle or to remove the thyroid from its passage.

A total of 28 patients have been injected over a 9 year period with a total of 131 injections. The average number was 6 with maximum 20 at time of writing. The average follow up time is 4.2 years.

The identified complications from the case note review are summarized in Table 1.

**Table 1:** Incidence of side effects following 131 injections of 28 patients of Longus Colli muscle over a 9 year period.


Dysphagia	6
Weak neck	4
Croaky voice	2
Vascular injury	0


## Discussion

### Are we actually targeting the LCo muscles?

In this study we successfully identified the muscle with EMG control between 22–28 mm deep. These measurements were made during the ultrasound study and the muscles identified by our ultrasound expert we are confident that this is indeed the longus colli. Most of the anterior neck tissue is retracted which accounts for the apparent reduced depth. In very long necks the lower fibres may be more laterally placed.

The addition of ultrasound imaging to the EMG procedure showed that with our technique, we are precisely targeting the LCo muscles. In this group of 7 patients the ultrasound confirms the reliability of LCo muscle injection using anatomical landmarks and EMG control alone as there is a good correlation between the localisation of the LCo under ultrasound and EMG control.

The additional use of ultrasound to the EMG guidance has been advised to target deep neck muscles and manage cervical dystonia complex cases [16], however to use this requires a different skill set, additional equipment, increased time and expense. As we have shown in this study that the LCo muscles are accurately identified we do not propose the additional use of ultrasound to inject the muscles.

### Are we at risk of damaging vascular structures?

By accessing the LCo from an anterior approach we are utilising the avascular corridor which is well known to neurosurgeons accessing the structures of the cervical spine via an anterior approach [11,12,13]. The vertebral arteries are placed posterolateral to the longus colli, however from C6–C1 they are encased in a bony canal in the transverse processes of the vertebral bodies. Below C6 the arteries are more laterally situated and are not at risk as long as a central approach is maintained [15]. Locating the cricoid cartilage at C6 and inserting the needle immediately next to the trachea avoids the carotid artery [17]. We have always been aware of the possibility of complications, however we have had no vascular injuries.

### Do we traverse the thyroid gland during the injection?

In picking the lower part of the neck to inject this does increase the likelihood of traversing the thyroid gland during the injection. This is particularly so in patients with shorter necks. During our 9 years of experience with this technique and additional injections in cricopharyngeus and the posterior cricoarytenoid muscles we have not seen any complication related to the thyroid. In the study the needle was seen to traverse the thyroid in 6/7 cases, this was not associated with any additional discomfort. It is standard clinical practice to investigate lesions of the thyroid with fine needle aspiration cytology, this is freely done in patients even if they are taking anticoagulant medication and has a low complication rate with 1% rate of haematoma formation [19]. It is also possible to do a core biopsy of the thyroid with a similar safety profile [20]. The needle electrode we use is 27 gauge which is the same size as the smallest FNA needles in use.

### Is the technique safe and effective?

One of the main concerns regarding injection of the LCo is dysphagia, which happened frequently with the initial approach to the muscle which was by direct injection through the mouth (personal communication MHM). The reason for this is the weakening of the upper oesophageal constrictor muscles lying between C1–C6 [18]. Dysphagia with this anterior cervical approach is not a common complication in our experience. Using our technique the tip of the needle is aimed below C6 therefore avoiding the pharyngeal constrictor muscles. We also check for activation on EMG of the cricopharyngeus before injection [4]. and move the needle to a lower position if it occurs. We divide the dose in two injection sites on each side of the LCo due to the length of the muscle with 30 u injected in each site, this allows a smaller dose in each site and reduces the risk of diffusion. Some patients do notice a transient increase in effort to swallow solids for up to 2 weeks post injection but none have required medical intervention to treat it and easily overcome the problem by taking a softer diet.

As previously noted [21,22], dysphagia is not uncommon following injections for cervical dystonia of any kind, even with due caution. However due to the severe disability anterocollis causes, we feel that the benefit outweighs the risk with this technique.

In one patient approximately a week following injection she presented to her local hospital in respiratory distress she was diagnosed with a lower respiratory tract infection. It was not clear that the LCo injection played a role in this problem as she was injected several times before and since without any complication.

We have not found the currently validated rating scales useful in anterocollis so we rate our patient’s improvement by assessing the ability to undertake activities of daily living and improvement in pain which they express as a percentage of normal. We realise that a validated rating scale would be useful.

Clinical manifestations were pulling of the head downward particularly provoked by walking carrying and eating, inability to hold the head up to watch tv or easily to talk to people. Most patients find it difficult to socialize or go out unaccompanied. After the injections the ability to do these things significantly improves.

The recoded benefit was between 0–100%, the majority of the patients not responding were patients with Parkinsonism and bent rigid neck. These patients are often sent to us as a last ditch attempt to improve their symptoms however we have not found the injections helpful for them in general. A few of the patients do derive benefit in terms of increased mobility and less pain but the posture does not correct. As a general rule we will inject these patients 1–3 times to see if they gain any benefit usually by mutual consent the injections are discontinued. If those injections are removed from the cohort and we consider the patients with dystonic anterocollis alone the benefit is between 60–100% with an average of 74%. We find that the benefit improves the longer the injections continue and most patients reach a plateau of persistent benefit including one patient who no longer needs to be injected.

## Conclusion

From this study and from our 9 years experience of the injection of LCo under EMG control using an anterior approach, we conclude that this technique is precise, safe and well tolerated. It’s also cost effective and beneficial to patients.
